# tANCHOR-cell-based assay for monitoring of SARS-CoV-2 neutralizing antibodies rapidly adaptive to various receptor-binding domains

**DOI:** 10.1016/j.isci.2024.109123

**Published:** 2024-02-05

**Authors:** Daniel Ivanusic, Josef Maier, Suheda Icli, Valeria Falcone, Hubert Bernauer, Norbert Bannert

**Affiliations:** 1 Sexually transmitted bacterial pathogens and HIV (FG18), Robert Koch-Institute, 13353 Berlin, Germany; 2 ATG:biosynthetics GmbH, 79249 Merzhausen, Germany; 3 Freiburg University Medical Center, Faculty of Medicine, Institute of Virology, University of Freiburg, 79104 Freiburg, Germany

**Keywords:** Biochemistry, Molecular biology, Immunology, Virology

## Abstract

Conventional neutralizing enzyme-linked immunosorbent assay (ELISA) systems for severe acute respiratory syndrome coronavirus 2 (SARS-CoV-2) mimic the protein-protein interaction between angiotensin-converting enzyme 2 (ACE2) and the receptor-binding domain (RBD). However, an easy and rapidly adaptative ELISA-based system for testing neutralizing antibodies against upcoming SARS-CoV-2 variants is urgently needed. In this study, we closed this gap by developing a tANCHOR-cell-based RBD neutralization assay that avoids time-consuming protein expression and purification followed by coating on ELISA plates. This cell-based assay can be rapidly adopted to monitor neutralizing antibodies (NAbs) against upcoming SARS-CoV-2 variants. We show that the results obtained with the tANCHOR-cell-based assay system strongly correlate with commercially available surrogate assays for testing NAbs. Moreover, this technique can directly measure binding between cell-surface-exposed RBDs and soluble ACE2. With this technique, the degree of antibody escape elicited by emerging SARS-CoV-2 variants in current vaccination regimens can be determined rapidly and reliably.

## Highlights

• Various SARS-CoV-2 receptor-binding domains (RBDs) are displayed on the cell surface
• Enables screening for neutralizing antibodies to SARS-CoV-2
• This approach allows testing for ACE2 binding on various RBDs
• No need for laborious protein purification because all steps are cell-based

## Graphical abstract

**Figure:**
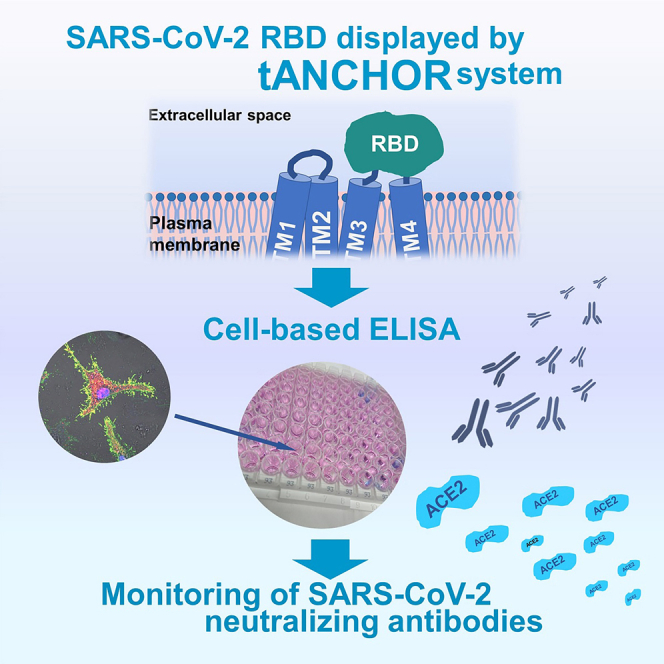

## Introduction

Severe acute respiratory syndrome coronavirus 2 (SARS-CoV-2) was the cause of an ongoing worldwide pandemic that originated in Wuhan (China) and continued with the appearance of several viral variants, from alpha to omicron.^1^^,^^2^ Humans can acquire immunity by infection and clearance of SARS-CoV-2 itself^3^ or by vaccination against the spike (S) protein.^4^^,^^5^^,^^6^^,^^7^ Until recently, all mRNA vaccine or vector regimens that efficiently reduce the risk of infection, severe disease, and death were based on the use of a spike sequence derived from the Wuhan and are adapted to the Omicron SARS-CoV-2 strain.^8^^,^^9^ The hallmark of RNA viruses is a high mutation rate that is beneficial for escaping neutralizing antibodies (NAbs). SARS-CoV-2 is an RNA virus in which mutations during replication and immune escape occur quickly.^10^ In particular, the receptor-binding domain (RBD) that interacts with the host protein angiotensin-converting enzyme 2 (ACE2) for cell invasion is constantly acquiring mutations.^11^ Fifteen of a total of thirty-seven mutations within the spike protein were identified within the Omicron B.1.1.529 RBD.^12^ Monitoring of immunoglobulin G (IgG) binding to the RBD with viral neutralization activity led to tests for antibody-mediated protection or vaccine efficiency. Upcoming SARS-CoV-2 variants can escape viral neutralization through mutation sites within the RBD.^13^ Enzyme-linked immunosorbent assay (ELISA) systems based on the antibody-RBD antigen interaction are not sufficiently accurate to monitor SARS-CoV-2 neutralizing activity because, in such assays, all classes of antibodies, e.g., IgG with lower neutralizing activity than avidity-matured IgGs, are detected on the same target.^14^ Measurement of the protein-protein interaction (PPI) between ACE2 and RBD that is blocked by NAbs offers higher accuracy for measuring the neutralization activity and a higher correlation with the plaque reduction neutralization test (PRNT). These ELISA-based virus neutralization assays use plates coated with purified RBD or ACE2 protein.^15^^,^^16^^,^^17^^,^^18^ However, an easy and rapidly adaptative ELISA-based system for testing NAbs against the RBD is needed. The global relevance of this topic has increased dramatically with the occurrence of upcoming variants of SARS-CoV-2. In the current study, we aimed to fill this gap by developing a cell-based neutralization assay that utilizes displayed SARS-CoV-2 RBD by the tANCHOR system and secreted soluble ACE2 (aa 1–740), including detection tags, to mimic the virus host PPI. The tANCHOR display system provides highly efficient and reliable presentation of heterologous proteins on the surface of human cells for antibody-binding studies and is based on the use of transmembrane domains (TMs) derived from the tetraspanin (Tspan) superfamily. Therefore, we choose this display system for anchoring the RBD on the cell surface in order to generate an antigen surface for testing of specific antibodies specifically directed against the RBD. A typical Tspan contains four TMs connected by intracellular, small, and large extracellular loops (ICL, SEL, and LEL).^19^ Importantly, the LEL is not necessary for routing a Tspan to the cell surface and can be replaced by heterologous protein sequences.^20^^,^^21^ In particular, the transmembrane anchors derived from the Tspan CD82 showed best performance for displaying proteins on the cell surface of human embryonic kidney 293T (HEK293T) or HeLa cells, where the protein of interest is fused and expressed as a chimeric-membrane-bound unit connected by optimized linker sequences between the third and fourth TM.^21^^,^^22^ For testing the neutralization activity of an SARS-CoV-2 variant, only the coding DNA sequence in the tANCHOR vectors has to be adjusted. This enables rapid adaptation to upcoming SARS-CoV-2 variants.

## Results

### Establishment of a cell-based ELISA for monitoring of specific SARS-CoV-2 RBD neutralizing antibodies

**Figure 1:**
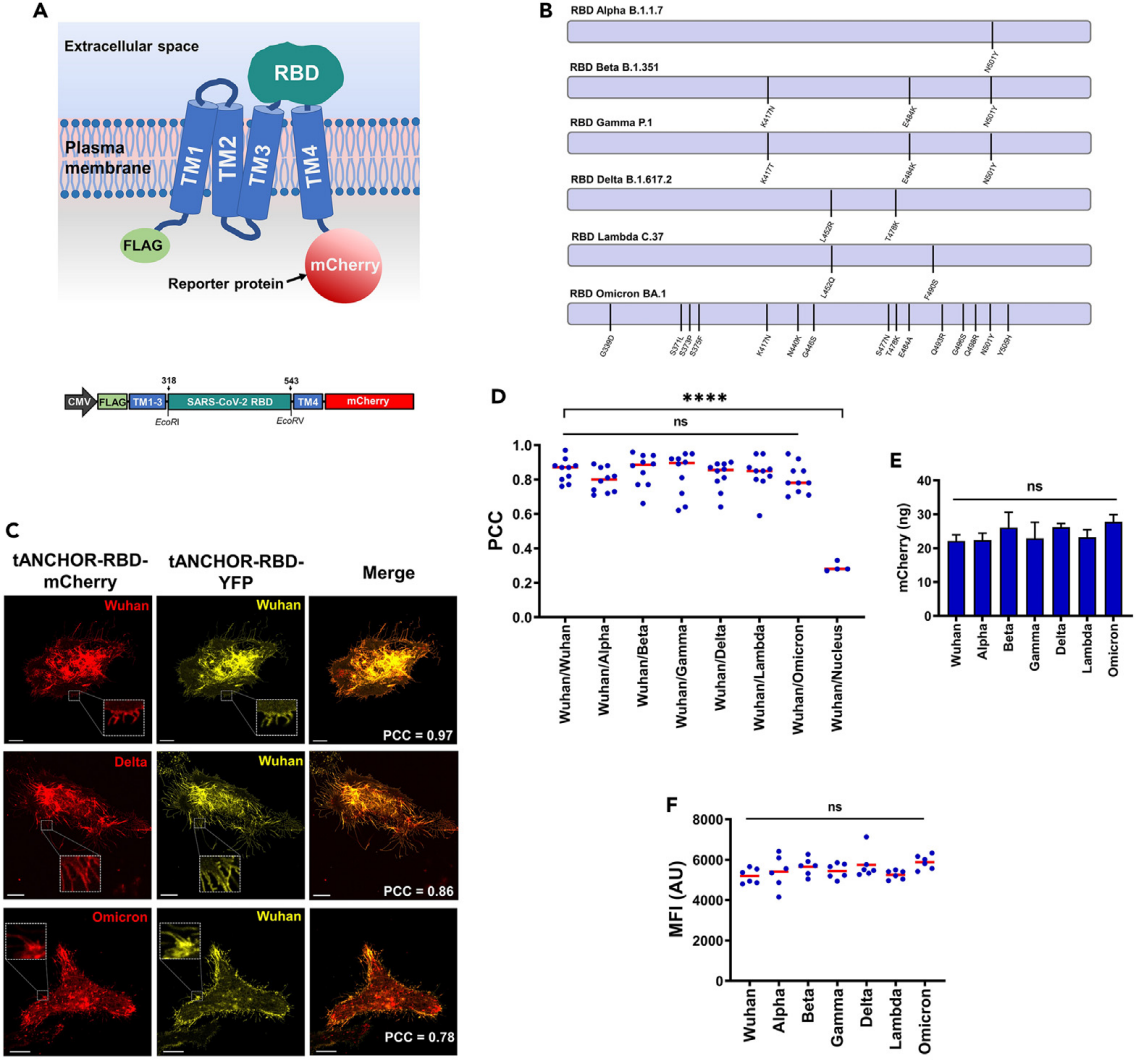
Development of a cell-based ELISA specific for SARS-CoV-2 RBD neutralizing antibodies (A) Cartoon of the topology of the expressed tANCHORed SARS-CoV-2 RBD and expression construct design (not to scale). TM: transmembrane helices 1–4 derived from CD82, mCherry: C-terminal red fluorescence protein used as a reporter protein, RBD: receptor-binding domain, FLAG: N-terminal tag DYKDDDDK. DNA coding for the RBD SARS-CoV-2 variants was inserted into the tANCHOR vector using the *Eco*RI and *Eco*RV restriction sites. (B) Illustration of the mutation sites within the RBDs. (C) Representative confocal laser scanning microscopy (CLSM) images of HeLa cells 48 h post-transfection expressing the tANCHORed RBDs of Wuhan, Delta, and Omicron fused with YFP, or mCherry, which was used for the calculation of Pearson’s correlation coefficient (PCC). Scale bars, 10 μm. See also Figure S1. (D) PCC calculation results (n = 10) showed no significant differences regarding protein localization between RBD variants and the ancestral Wuhan RBD. (E) Quantification of mCherry reporter protein (n = 4) indicates no significant effect of variant-specific RBD mutations on protein expression or (F) mCherry mean fluorescence intensity (MFI) (n = 6). (D–F) Significance was estimated by one-way ANOVA; non-significant (ns): >0.05; ∗p < 0.05, ∗∗∗∗p < 0.0001. AU, arbitrary units.

We developed a cell-based ELISA for rapid and easy adaptation to upcoming SARS-CoV-2 variants by employing the tANCHOR protein display system.^21^ This display system utilizes transmembrane anchors derived from Tspan sequences. Protein expression can be followed-up by fusion to the mCherry reporter protein (Figure 1A). The hallmark of the tANCHOR system, especially for CD82-derived transmembrane domains, is its high efficiency for displaying proteins or peptides on the surface of human cells.^21^ Therefore, we used this system to display the receptor-binding domain (RBD) of the Alpha (B.1.1.7), Beta (1.351), Gamma (P.1), Delta (B.1.617.2), Lambda (C.37), Omicron (BA.1), and the ancestral Wuhan (Wuhan-Hu-1) strain of SARS-CoV-2. Spike sequences corresponding to the amino acids (aa) 318–543 of the Wuhan-Hu-1 strain were defined as individual RBDs (Figure 1B). They were synthesized and inserted instead of the large extracellular loop (LEL) of the Tspan CD82 within the tANCHOR vector using the restriction sites *Eco*RI and *Eco*RV (Figure 1A). This procedure enables us to rapidly generate RBD display constructs (tANCHORed RBDs) for any upcoming variant of SARS-CoV-2.

### Expression and validation of tANCHORed SARS-CoV-2 RBD variants

Introduction of mutation sites within the RBD may influence the localization and expression level of tANCHORed RBDs. In order to identify such effects, we first coexpressed the ancestral Wuhan tANCHORed RBD fused with YFP together with the Wuhan, Alpha, Beta, Gamma, Delta, Lambda, and Omicron tANCHORed variant RBD fused with mCherry by transient transfection in HeLa cells. The colocalization of the tANCHORed Wuhan RBD fused to YFP was observed for all SARS-CoV-2 tANCHORed RBD variants fused to mCherry (Figures 1C and S1). We used the fluorescence intensities of YFP and mCherry to calculate the Pearson correlation coefficient (PCC) for each pair. We observed no significant differences in the protein localization of the different tANCHORed RBDs (Figure 1D). Further, we quantified the expressed protein using the mCherry reporter (Figure 1E) and measured the emitted mean fluorescence intensity (MFI). We found no significant differences in the protein expression level between the variants compared with the Wuhan RBD (Figure 1F). Taken together, our data indicate that the RBD display using the tANCHOR system provides a robust and similar protein localization and expression level.

### Stability of the cell monolayer during washing steps

**Figure 2:**
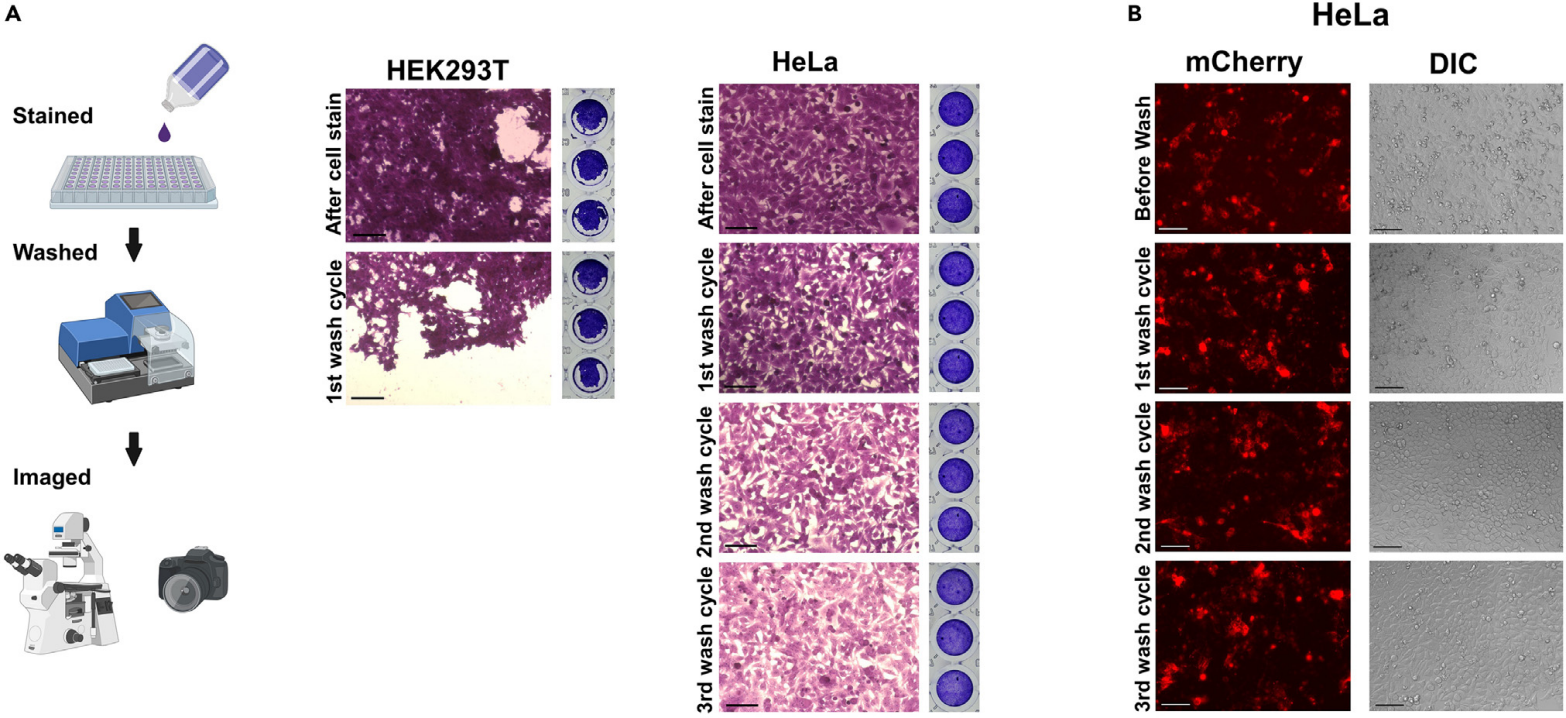
Analysis of cell adherence during washing steps (A) Experimental steps for testing cell adherence during plate washing (left). Images of HEK293T or HeLa cell layers after staining with crystal violet and washing with an ELISA washer. (B) HeLa cells 48 h post-transfection with 0.3 μg plasmid DNA (ptANCHOR-CD82-Wuhan-mCherry) per well of a 96-well plate. Cell layers were fixed using 2% paraformaldehyde (PFA) as a fixative when tested for cell adherence during washing steps. Scale bars, 100 μm.

Next, we tested if a cell monolayer could withstand washing steps using an automated ELISA washer because any ELISA system requires an automated ELISA washer when screening of large sample sets is desired. For this purpose, we used HEK293T and HeLa cells, which are routinely utilized for cell-based techniques.^23^^,^^24^^,^^25^ The cells were stained with crystal violet and followed-up the integrity of cell monolayers by microscopy and images of the 96-well plate bottom (Figure 2A). We observed that the staining and washing steps greatly affected the integrity of 293T monolayers. By contrast, HeLa cell monolayers were still intact even after five washing steps, and crystal violet in particular was washed out of the cells (Figure 2A). Even if HeLa cells are transfected with the plasmid ptANCHOR-CD82-Wuhan-mCherry, we have observed that the cell layers are still intact (Figure 2B). We intentionally did not use cell culture surface coatings such as poly-L-lysine for enhanced cell adherence because this step would make the assay laborious before cells could be seeded. The ability to use an ELISA washer with a low flow rate during cell-based ELISA techniques will enable the testing of large numbers of sera for neutralizing activity.

### Secretion, characterization, and optimization of ACE2-V5-His binding conditions

**Figure 3:**
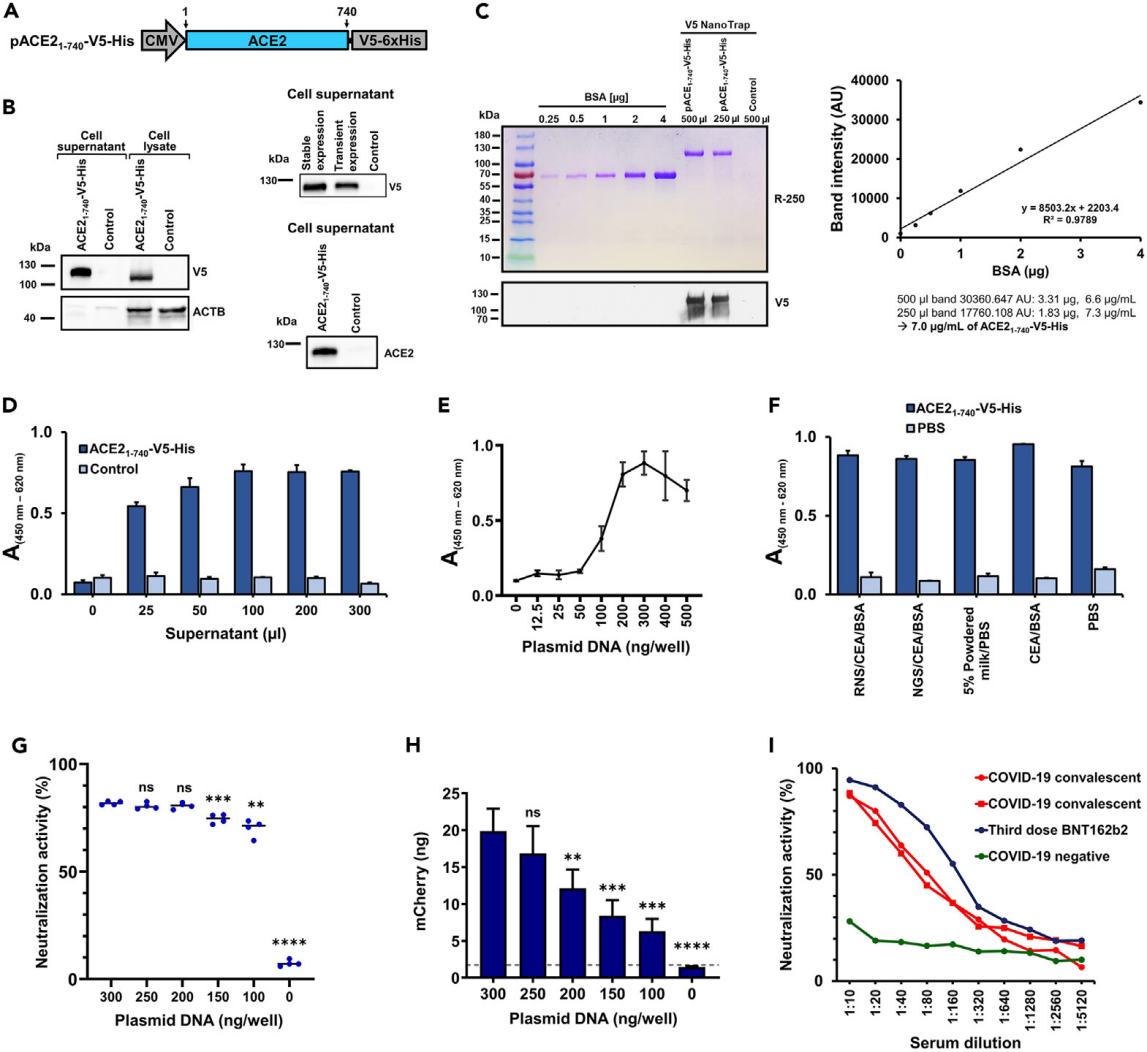
Characterization and binding analysis of soluble ACE2_1-740_-V5-His (A) Illustration of the ACE2_1-740_-V5-His expression construct (not to scale). ACE2: angiotensin-converting enzyme 2, V5-6xHis: C-terminal tag GKPIPNPLLGLDST fused with 6xhistidine. (B) ACE2_1-740_-V5-His transient protein expression in HEK293T cells (left) analyzed by western blot analysis using antibodies directed against the V5 epitope. Comparison of protein expression between transiently transfected HEK293T cells and the stable HEK293T cell line (right, top). Secreted ACE2_1-740_-V5-His protein from HEK293T stable cell line was probed with antibodies directed against human ACE2 protein (right, bottom). (C) Quantification of secreted ACE2_1-740_-V5-His protein obtained from a stably transfected HEK293T cell line. Bovine serum albumin (BSA) was used to generate a standard curve to identify the amount of secreted protein isolated by a V5 trap from the collected supernatant. Control contains supernatant from non-transfected HEK293T cells. (D) Determination of the supernatant amount for obtaining the highest absorbance value on the Wuhan RBD (HeLa cells transfected with 0.3 μg plasmid DNA per well). (E) Titration of the plasmid DNA amounts to reach the highest possible absorbance value for HeLa cells expressing the Wuhan RBD. (F) Testing of different blocking conditions using HeLa cells expressing the Wuhan RBD on the cell surface (HeLa cells transfected with 0.3 μg plasmid DNA per well). RNS, rabbit normal serum; NGS, normal goat serum; CEA, chicken egg albumin; BSA, bovine serum albumin; PBS, phosphate-buffered saline. (G) Results of cell-based neutralization assays (n = 4) using different plasmid DNA amount of ptANCHOR-CD82-Wuhan-mCherry. (H) Quantification of expressed mCherry fusion proteins (n = 4) from experiment (G). Expressed protein was collected for each quantification data point from three wells by cell lysis. (I) Impact on neutralization activity using different serum dilutions; each neutralization activity data point represents the mean of results from two wells. In each 96-well, 100 μL (0.7 μg protein) of ACE2_1-740_-V5-His-containing supernatant was used for performing experiments (E)–(G), and (I). Groups are compared for significance to the highest plasmid DNA amount used in the experiment by an unpaired two-tailed Student’s t test; non-significant (ns): >0.05; ∗∗p < 0.01, ∗∗∗p < 0.001, ∗∗∗∗p < 0.0001.

We cloned the coding sequence of the human ACE2 protein for expression of the amino acids (aa) 1–740 fused to V5-6xHis tag in the expression vector FlexMam-Puro and named the vector pACE2_1-740_-V5-His (Figure 3A). This truncated version of the ACE2 ectodomain (aa 1–740) is known to be secreted into the cell culture medium.^26^ Secreted ACE2_1-740_-V5-His protein was detectable in the supernatant and in the cell lysates of transiently transfected HEK293T cells (Figure 3B, left). Generation of a HEK293T-based stable cell line producing ACE2_1-740_-V5-His showed stable and robust secretion of ACE2_1-740_-V5-His protein (Figure 3B left, top) that can be probed with antibodies against human ACE2 protein (Figure 3B, bottom). We further quantified the secreted ACE2_1-740_-V5-His protein from a pooled batch and obtained 7.0 μg/mL protein in the supernatant, which was isolated by a V5-nanoTrap, blotted to a membrane after staining with Coomassie dye, and probed with antibodies directed against the V5 epitope (Figure 3C). Next, we tested how much supernatant containing ACE2_1-740_-V5-His protein is necessary to obtain the highest possible absorbance value when binding was measured to the Wuhan RBD in our 96-well plate format. To this end, we transfected HeLa cells and tested different amounts of supernatant obtained from the stable cell line secreting ACE2_1-740_-V5-His protein. We observed that binding to the expressed RBD on the cell surface is saturated at an applied volume of 100 μL to the wells, which corresponds to 0.7 μg ACE2_1-740_-V5-His protein (Figure 3D). A correction for background can be achieved by subtracting the absorbance obtained without adding ACE2_1-740_-V5-His from results where the receptor protein was added. The plasmid DNA amount that produced the highest absorbance value was also titrated by transient transfection with the plasmid ptANCHOR-CD82-Wuhan-mCherry. We found that a suitable DNA amount for transfection of HeLa cells with 1.0 μL of the transfection reagent are 300 ng per well of a 96-well plate (Figure 3E). Of note, cells were starting to detach when more than 300 ng/well was used for transfection. This is a sign of cell death that leads to lower amounts of ACE2_1-740_-V5-His binding.

We further optimized the binding assay of soluble ACE2_1-740_-V5-His to RBDs displayed on the surface of HEK293T cells by reducing background binding. We observed that applying a blocking solution containing 2% chicken egg albumin (CEA), 3% bovine serum albumin fraction V (BSA), and 10% normal goat serum (NGS) in 1X PBS (phosphate-buffered saline) produced the lowest background in the developed cell-based ELISA (Figure 3F).

A key application of our cell-based binding assays is neutralization ELISA in which the binding of ACE2_1-740_-V5-His to the RBD is blocked by antibodies in sera or plasma dilutions.^27^^,^^28^ The interaction platform with ACE2 is located within the RBD that is exposed on the SARS-CoV-2 full-length envelope spike protein. Our *in vitro* cell-based assay mimics the naturally occurring protein-protein interaction at the entry step of the SARS-CoV-2 spike protein with the host cell receptor ACE2.^29^ In order to evaluate how a difference in RBD expression levels influences the outcome of neutralization assays, we transfected HeLa cells with 0, 100, 150, 200, 250, and 300 ng of ptANCHOR-CD82-Wuhan-mCherry. Protein expression was assayed using the mCherry reporter and showed the expected correlation with the transfected amount of ptANCHOR-CD82-Wuhan-mCherry plasmid (Figure 3G). The obtained neutralization results at various tANCHORed RBD expression levels assayed with a convalescent serum as a source of NAbs were comparable (Figure 3H). This indicates that less difference in RBD expression levels does not significantly influence the neutralization result.

Next, we evaluate the serum dilutions of two convalescent individuals as well as a serum from a multiple vaccinated person and a pre-pandemic serum. As expected, the neutralizing activity decreases with increasing dilution and allows the determination of the dilution with 50% neutralization activity. (Figure 3I). Notably, a serum dilution of 1:10 produced an undesired background of measured neutralization activity when COVID-19 negative serum was used. An appropriate dilution to start with to test patient serum is therefore 1:20.

### Binding of soluble ACE2_1-740_-V5-His protein on various SARS-CoV-2 RBD variants

**Figure 4:**
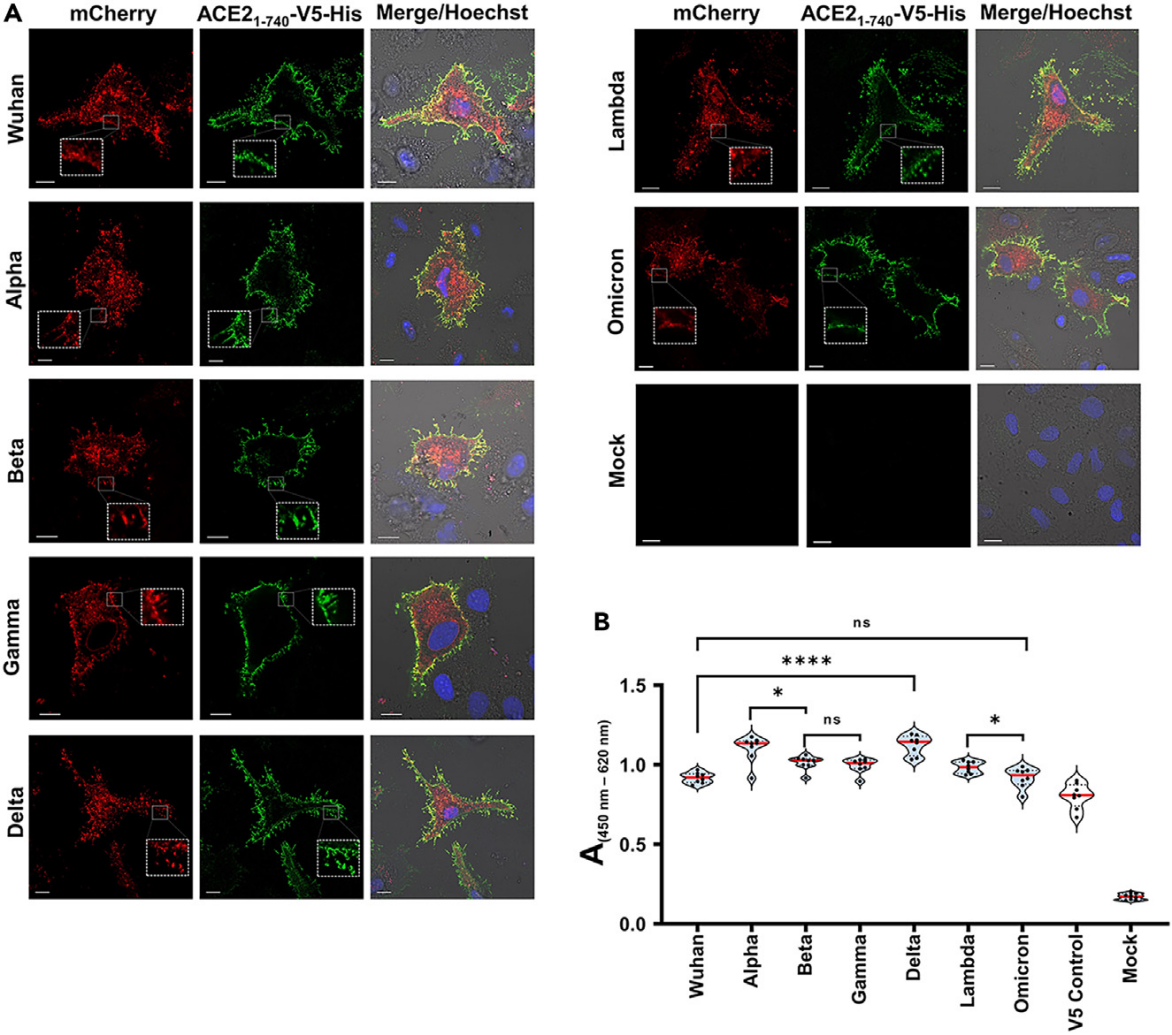
Binding of ACE2_1-740_-V5-His on various SARS-CoV-2 RBD variants (A) Representative confocal laser scanning microscopy (CLSM) images of immunostained ACE2_1-740_-V5-His protein (green) bound on transfected HeLa cells expressing tANCHORed RBD protein (red); scale bars: 10 μm. Mock control contains transfected HeLa cells without plasmid DNA and incubated with ACE2_1-740_-V5-His protein in parallel. Images were merged and show nuclei stained with Hoechst 33342 (blue), DIC (differential interference contrast), and fluorescence channels. (B) Violin plots of the ACE2_1-740_-V5-His protein binding to RBD variants (n = 8). The V5 control (HeLa cells transfected with ptANCHOR-CD82-V5-His-mCherry) was not incubated with a supernatant containing secreted ACE2_1-740_-V5-His protein. The p values were calculated by an unpaired, two-tailed Student’s t test; non-significant (ns): >0.05; ∗p < 0.05, ∗∗∗∗p < 0.0001.

The bound ACE2_1-740_-V5-His protein was visualized by indirect immunostaining on the cell surface of HeLa cells expressing the tANCHORed RBD of the Wuhan, Alpha, Beta, Gamma, Delta, Lambda, and Omicron variants. All displayed RBD were able to bind to the secreted ACE2_1-740_-V5-His protein (Figure 4A). Further, we tested differently transfected HeLa cells, which express one of the tANCHORed RBDs of Wuhan, Alpha, Beta, Gamma, Delta, Lambda, and Omicron SARS-CoV-2, respectively, in parallel assays for the binding efficiency of soluble ACE2 on the cell surface. This approach enables comparing the binding characteristics of ACE2 and the RBD variants in parallel. We observed a significant difference between the Wuhan and Delta and no significant differences between the Omicron and the Wuhan RBDs or between the Beta and Gamma RBDs. Additional significant differences were detected between the Alpha and Beta RBDs and between the Lambda and Omicron RBDs (Figure 4B). This comparison demonstrates that mutation sites within the RBD have an impact on the binding affinity of the ACE2 protein. For upcoming variants, this assay can therefore be used to characterize the interaction between the viral RBD and ACE2, which is useful to rate the infection profile of SARS-CoV-2.

### Testing of sera from different groups by using the developed tANCHOR-cell-based ELISA for measuring neutralizing activity

**Figure 5:**
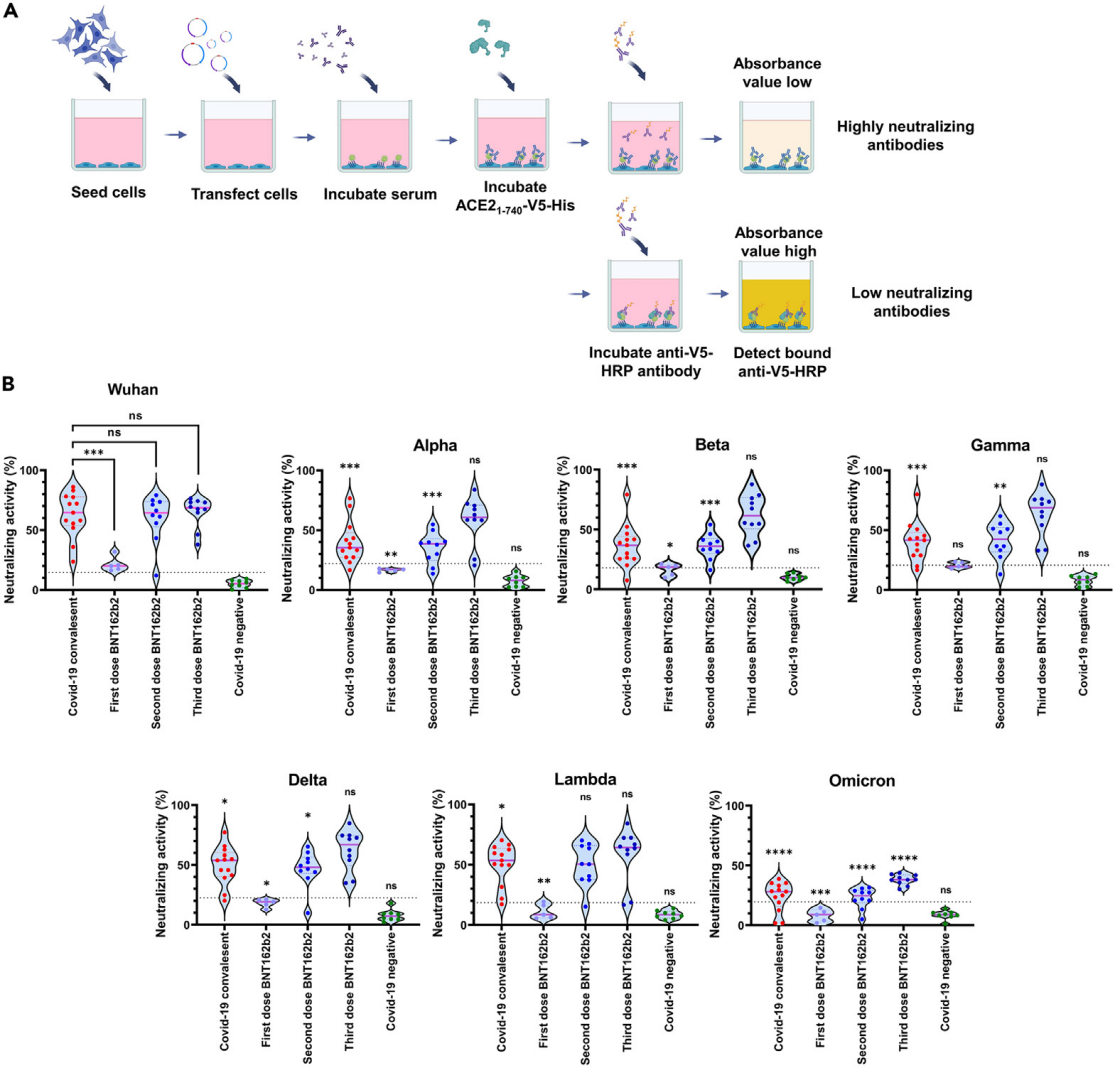
tANCHOR-cell-based RBD ELISA for detecting neutralizing antibodies in different serological groups (A) The cartoon illustrates the workflow for the developed cell-based ELISA. (B) Results of using the developed cell-based ELISA to analyze human serum for neutralizing activity against the displayed Wuhan, Alpha, Beta, Gamma, Delta, Lambda, and Omicron RBD for SARS-CoV-2 RBD-specific NAbs. The neutralizing activity values were determined at 1:20 serum dilution. The dotted line represents the cutoff. The p value was calculated by an unpaired two-tailed Student’s t test groupwise between Wuhan and other variants and within the Wuhan RBD; the significance is indicated on connection lines between the compared groups. non-significant (ns): >0.05; ∗p < 0.05, ∗∗p < 0.01, ∗∗∗p < 0.001, ∗∗∗∗p < 0.0001.

For performing the optimized neutralization assay, HeLa cells are seeded in a 96-well format and transfected in order to display the RBD of choice on the cell surface, offering an interaction platform for ACE2. Incubation of serum from convalescent or vaccinated individuals will lead to binding of antibodies to the RBD, and free binding sites within the RBD that are not masked by NAbs are then detected by incubation with soluble ACE2_1-740_-V5-His protein. A low binding of ACE2_1-740_-V5-His will indicate the presence of NAbs. By contrast, highly efficient binding of ACE2_1-740_-V5-His will mean that antibodies are not able to block the interaction between the ACE2- and RBD-binding sites (Figure 5A). Using a panel of convalescent sera (n = 13), from individuals receiving one (n = 5), two (n = 10), or three (n = 10) doses of Pfizer-BioNTech BNT162b2 mRNA vaccine and COVID-19-negative sera (n = 8), we demonstrate that there are different potencies of neutralizing activity when different RBD variants are used for the neutralization assay (Figure 5B). We observed that the neutralization profile against all RBD variants, except the Omicron RBD, by antibodies derived from a three-vaccination schedule with BNT162b2 is comparable with the neutralization activity where the Wuhan RBD was used. Interestingly, all tested sera derived from convalescent individuals using the Wuhan RBD showed lower neutralization activity compared with the Alpha, Beta, Gamma, Delta, and Omicron RBD variants. An extremely low neutralization activity was observed overall when the Omicron RBD was used. The highest neutralizing activity was measured for individuals receiving three doses of BNT162b2 mRNA vaccine, with a mean value of 65.0% neutralizing activity for the Wuhan RBD. By contrast, the mean neutralizing activity value in the convalescent group was 62.8%. As expected, two doses of BNT162b2 resulted in the same neutralization profile for the convalescent group as observed by other reports.^30^^,^^31^

### Correlation of the developed tANCHOR-based ELISA assay with a commercially available neutralizing ELISA system

**Figure 6:**
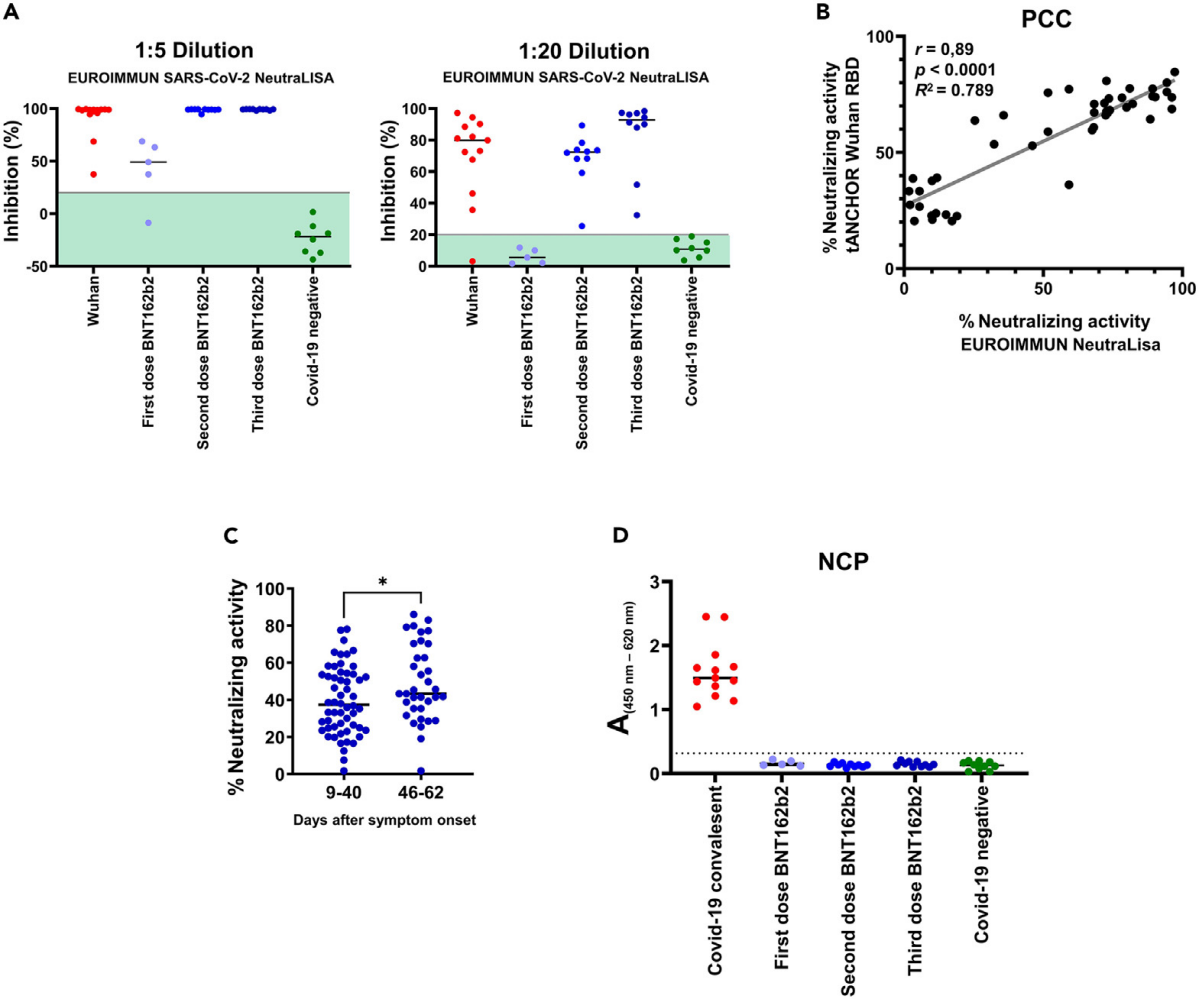
Validation of the developed cell-based ELISA (A) Sera was analyzed employing a commercially available neutralization assay (NeutraLISA assay from EUROIMMUN) at a dilution of 1:5 and 1:20. The range for negative specimens defined by the manufacturer is displayed as pale green background. (B) Comparison of neutralizing values obtained from NeutraLISA and tANCHOR-cell-based ELISA inhibition results in the full sample subset, Pearson’s correlation coefficient (PCC). (C) Comparison of neutralization activity obtained for the Wuhan, Alpha, Beta, Gamma, Delta, Lambda, and Omicron RBDs between the early and late stages of SARS-CoV-2 infection. (D) Testing for antibodies against the nucleocapsid protein (NCP) for detection of SARS-CoV-2 infection in the used serum panel and two pre-pandemic controls. The dotted line represents the cutoff value; ∗p < 0.05.

Next, we used the same serum panel to compare the developed SARS-CoV-2 neutralization assay with a surrogate ELISA assay similarly based on blockage of the PPI between ACE2 and the RBD, which is commercially available for testing NAbs (NeutraLISA, EUROIMMUN). This *in vitro* test produces results that are strongly correlated with plaque reduction neutralization tests (PRNT) using replication-competent SARS-CoV-2 particles.^32^^,^^33^ Unfortunately, this assay is only available for the Wuhan variant; therefore, we were only able to compare our neutralizing data for the Wuhan RBD. We performed the NeutraLISA assay as recommended by the manufacturer at a dilution of 1:5. However, testing the sera at a dilution of 1:20 allowed for better differentiation of the neutralization activity between the different groups (Figure 6A), and a high correlation (*r* = 0.89) between the tANCHOR-cell-based ELISA and the EUROIMMUN NeutraLISA assay was observed (Figure 6B). Further, we compared antibodies from convalescent individuals at an early stage (9–40 days after symptom onset) and a later stage (46–62 days after symptom onset) of infection and observed, for all RBD variants, higher neutralization activity at later infection stages (Figure 6C). We compared these two groups because IgG titers reach a plateau six weeks after symptom onset, and IgM titers decline between two and five weeks post-symptom onset.^34^ Additionally, ELISA performed against the SARS-CoV-2 nucleocapsid protein confirmed that only the convalescent group was literally infected with the pandemic SARS-CoV-2 virus (Figure 6D). In particular, testing antibodies directed against the nucleocapsid can differentiate between infected and vaccinated individuals.^35^ The sera obtained from vaccinated individuals in our sample subset therefore contained only antibodies raised against the RBD that were induced by the spike proteins expressed from the mRNA vaccine.

## Discussion

The initial outbreak of the SARS-CoV-2 pandemic has evolved into a world of variants where the ancestral SARS-CoV-2 strain, with its origin in Wuhan, China, has been outcompeted. This is the result of ongoing selection of SARS-CoV-2 variants that can replicate efficiently and evade the immune system. The target of most neutralizing antibodies (NAbs) produced by the immune system is the receptor-binding domain (RBD), which is essential for cell entry.^27^^,^^36^^,^^37^ This fatal infection step is critical for the virus; therefore, most selected mutation sites can be found within the RBD.^38^ Induced antibodies against the RBD bind to a specific epitope, and mutations within the epitope may enable the virus to escape the immune system. It is necessary to screen for NAbs against upcoming variants in order to surveil for SARS-CoV-2 variants that cannot be neutralized with antibodies induced by infection of a former circulating viral variant or, more importantly, vaccination regimes. The gold-standard technique for monitoring NAbs is the plaque reduction neutralization test (PRNT). However, this test requires the handling of an SARS-CoV-2 replication-competent virus in a BSL3 laboratory facility by well-trained staff and is time-consuming.^39^^,^^40^ Pseudotyped-virus-based assays require BSL-2 conditions, but this method is not suitable for high-throughput screening (HTS) and cannot be rapidly adapted to upcoming variants.^41^^,^^42^ Therefore, neutralization assays that are suitable for HTS without the use of infectious virus preparation are still needed. These are the reasons why ELISA-based assays mimicking the ACE-RBD interaction were developed. These ELISA systems use ELISA plates coated with purified RBD protein to test recombinant human ACE2 residual interaction sites in the presence of sera.^15^^,^^16^^,^^17^^,^^18^ There are some commercially available assays that can measure NAbs *in vitro*. However, these assays cannot be rapidly adjusted to upcoming circulating variants because the development of a new ELISA system always requires the expression and purification of the RBD of the upcoming variant and the coating of the ELISA plate with this protein. This is obviously time-consuming, and companies are uneasy about investing financial resources in a new ELISA system because, in the coming months, an upcoming variant can potentially appear. A cell-based ELISA was developed, and the use of cells offering an ACE2 reaction surface for purified RBD protein was shown to be possible.^43^ However, this assay cannot be rapidly adapted to the currently circulating SARS-CoV-2 variants because the RBD must be expressed and purified for a PPI test on Vero cells that express human-like ACE2. To avoid the need to express and purify a new RBD every time when a variant appears, we developed the tANCHORed-cell-based ELISA. The major advantage of this approach is that the transfected cells display the RBD on the surface, offering a PPI platform for ACE2 testing. Moreover, there is no need to purify the secreted ACE2_1-740_-V5-His from the supernatant. The generated stable cell line provides an inexpensive source of the soluble ACE2 interaction component for testing neutralizing antibodies directed against the RBD. Therefore, for all assay components, no additional isolation or purification step is necessary. Another advantage of our developed assay is that the interaction between ACE2 and the RBD can be directly compared. The only factor that influences this approach is the equal amount and quality of plasmid DNA. In line with other findings, we were able to confirm that the Omicron variant binds with equal affinity to ACE2 as reported for the Wuhan RBD; the highest binding affinity was observed on the Delta variant; and variants containing the mutation site N501Y in the Alpha, Beta, and Gamma RBD enhance ACE2-binding affinity (Figure 4B).^44^^,^^45^ With none of the displayed RBD variants we did not observe any mislocalization or extreme differences in protein expression level (Figures 1E and 1F). For testing explicitly neutralizing activity in sera, our surrogate assay allows some degree of discrepancy in the displayed RBD protein amount (Figures 3G and 3H). This was expected because the reference for calculating neutralization activity is always the absorbance value at saturated binding of the displayed RBD variant by ACE2_1-740_-V5-His. This consideration is relevant because a different variant could possibly cause an altered expression of its tANCHORed RBD. In contrast, when ACE2 binding has to be measured and compared between RBD variants, the RBD-protein expression level must be additionally normalized. Although the transient transfection procedure is suitable for different variants where rapid results are needed, this developed assay should go a step further by generating stable cell lines expressing the indicated RBDs if time permits. Stable cell lines will certainly reduce the costs for performing the SARS-CoV-2 neutralization tests and will avoid transfection optimization. It is important to acknowledge that the PRNT evaluates not only RBD-targeting neutralizing antibodies but also non-RBD-targeting antibodies, emphasizing the need for comprehensive consideration.^46^^,^^47^^,^^48^ Our assay can support the PRNT in case of a prescreening method and more importantly, to compare neutralization of RBD-targeting and non-RBD-targeting neutralizing antibodies by using data from PRNT results. In addition, gain-of-function (GoF) research focused on the RBD variants that most efficiently escape the immune system in combination with increased ACE2 affinity can be performed without the risk of developing an infectious virus that can be potentially extremely pathogenic to humans.

### Limitations of the study

There are certain limitations to the current study that require attention. One of the general limitations of the study that needs to be addressed is the principle of measuring the blockage of the interaction between the RBD and ACE2. Other possible neutralizing antibodies that are directed against the S2 domain or outside of the RBD region will not be detected by our developed cell-based neutralization assay. We cannot rule out the possibility that for upcoming SARS-CoV-2 variants, neutralizing antibodies outside the RBD region will become more important to estimate the neutralization activity. Additionally, we did not include any serum derived from SARS-CoV-2-asymptomatic individuals. So, the neutralization activity measured by the cell-based assay of those individuals is not known and should be addressed in future applications.

## STAR★Methods

### Key resources table

**Table:** 

REAGENT or RESOURCE	SOURCE	IDENTIFIER
**Antibodies**		

Mouse anti-V5-HRP	Invitrogen by Thermo Fisher Scientific	Cat# 46-0708
Mouse anti-beta-actin-HRP	Invitrogen by Thermo Fisher Scientific	Cat# MA5-15739-HRP
Rabbit anti-ACE2	Invitrogen by Thermo Fisher Scientific	Cat# MA-32307
Rabbit anti-V5	Novus Biologicals	Cat# NB600-381
goat anti-rabbit Alexa-Fluor 488	Invitrogen by Thermo Fisher Scientific	Cat# A11034
Rabbit anti-human-HRP	Dako	Cat# P0214
Goat anti-rabbit-HRP	Dako	Cat# P0448

**Bacterial and virus strains**		

Chemocompetent *E. coli* DH5α	New England Biolabs	Cat# C2987H

**Biological samples**		

Human serum	University Medical Center, Freiburg	N/A

**Chemicals, peptides, and recombinant proteins**		

SARS-CoV-2 nucleoprotein NCP (aa 2–419)	Miltenyi Biotec	Cat# 130-127-462
*Eco*RI-HF	New England Biolabs	Cat# R3101
*Eco*RV-HF	New England Biolabs	Cat# R3195
Q5 Hotstart High-Fidelity DNA Polymerase	New England Biolabs	Cat# M0493
*Pme*I	New England Biolabs	Cat# R0560
*Cla*I	New England Biolabs	Cat# R0197
*Nhe*I-HF	New England Biolabs	Cat# R3131
T4 DNA Ligase	New England Biolabs	Cat# M0202
Benzonase Nuclease	Millipore	Cat# 70746-10KU
PFA	Carl Roth	Cat# 0335.1
Bovine Serum Albumin Fraction V (BSA)	Carl Roth	Cat# T844.2
Chicken egg albumin (CEA)	Sigma Aldrich	Cat# A5253
Normal goat serum (NGS)	Biowest	Cat# S2000-500
Normal rabbit serum (NRS)	Biowest	Cat# S2500-500
DMEM medium high glucose	Robert Koch-Institute, inhouse preparation	Order No# 8
1x Phosphate-buffered saline (PBS)	Robert Koch-Institute, inhouse preparation	Order No# 27
Fetal bovine serum (FBS)	Gibco	Cat# 11573397
Penicillin-Streptomycin	Gibco	Cat# 15140122
Puromycin	Carl Roth	Cat# 0240.4
Trypsin-EDTA	Gibco	Cat# 25200056
Hoechst 33342 stain	Immunochemistry	Part# 639
Metafectene	Biontex	Cat# T020
Bovine serum albumin (BSA) standard 2 mg/mL	Thermo Scientific	Prod# 23209
Nanobody/VHH V5-Trap magnetic agarose	Chromotek	Cat# V5tma
Dynabeads His-Tag Isolation and Pulldown	Invitrogen by Thermo Fisher Scientific	Cat# 10103D
Milk powder blotting grade	Carl Roth	Cat# T145.3
Tween-20	Sigma Aldrich	Car# 93773
Halt Protease inhibitor cocktail 100x	Thermo Scientific	Cat# 87786
Crystal violet	Carl Roth	Cat# T123.3
Methanol	Carl Roth	Cat# 4627.2
Glacial acetic acid (100%)	Carl Roth	Cat# 3738.5
TMB (3,3′,5,5′-tetramethylbenzidine) 11x and TMB buffer	BioRad	Cat# TMB7BR
2M H_2_SO_4_ TMB stopp solution	BioRad	Cat# STP1BR
Glycine	Carl Roth	Cat# 3187.5
Tris	Carl Roth	Cat# 0188.3
NaCl	Carl Roth	Cat# 3957.2
Na_3_PO_4_	Carl Roth	Cat# T107.1
Imidazole	Carl Roth	Cat# X998.4
NaHCO_3_	Merck	Cat# 106329.1000
Na_2_CO_3_	Merck	Cat# 106392.1000
Coomassie Brilliant Blue R-250	Carl Roth	Cat# 3862.2
SuperSignal West Pico PLUS substrate	Thermo Scientific	Cat# 34577
2x Laemmli buffer	Sigma Aldrich	Cat# S340-1VL
NaOH 2M	Carl Roth	Cat# T135.1
Agarose	Thermo Scientific	Cat# 16500-500
Pierce BSA standard 2 mg/mL	Thermo Scientific	Cat# 23209

**Critical commercial assays**		

mCherry Quantification kit	Abcam	Cat# AB284566
NeutraLISA	EUROIMMUN	Cat# EI 2606-9601-4
MaxiPrep kit	Qiagen	Cat# 12163
QIAprep Spin Miniprep	Qiagen	Cat# 27106

**Experimental models: Cell lines**		

HeLa	ATCC	CCL-2
HEK293T	ATCC	CRL-3216

**Oligonucleotides**		

YFP-*Cla*I for: Table S2	Integrated DNA Technologies	N/A
YFP-*Pme*I rev: Table S2	Integrated DNA Technologies	N/A

**Recombinant DNA**		

Gene synthesis fragments: Table S1	ATG:biosynthetics GmbH	N/A
ptANCHOR-CD82-V5-His-mCherry	ATG:biosynthetics GmbH	Cat# TA-CDA-ASA1-V5-His
FlexMam-Puro	ATG:biosynthetics GmbH	Cat# TA-CDA-ASA1-Puro
pCMV-CD63-YFP	Ivanusic et al.^49^	N/A

**Software and algorithms**		

GraphPad Prism 9.2.0	Graphpad Software Inc	www.graphpad.com
ZEISS ZEN Blue Edition 3.4	Carl Zeiss AG	www.zeiss.de
Microsoft Office Professional 2019	Microsoft	www.microsoft.com
Fiji	Open-source platform	http://fiji.sc
Images partially created with BioRender.com	BioRender	https://biorender.com/

**Other**		

96-well tissue culture testplate 96F	TPP	Cat# 92096
96 optical well plate	Greiner Bio-One	Cat# 655101
6 well tissue culture plate	TPP	Cat# 92006
Tissue cell culture flask 300	TPP	Cat# 90301
Tissue cell culture flask 150	TPP	Cat# 90151
Tissue cell culture flask 75	TPP	Cat# 90076
Tissue cell culture flask 25	TPP	Cat# 90026
Nunc MaxiSorp assay plates	Thermo Scientific	Cat# 442404
μ-slide 8 well high glass bottom	IBIDI	Cat# 80807
ROTILABO ELISA seal film	Carl Roth	Cat# EN76.1

### Resource availability

#### Lead contact

Further information and requests for resources and reagents should be directed to and will be fulfilled by the lead contact, Dr. Daniel Ivanusic (Daniel.Ivanusic@web.de).

#### Materials availability

Plasmids containing the patented tANCHOR technology generated in this study will be made available on request to ATG:biosynthetics GmbH, but payment and a completed Materials Transfer Agreement may be required.

#### Data and code availability

• The datasets generated and analyzed in this study will be shared by the lead contact upon reasonable request.
• This paper did not generate any code.
• Any additional information required to reanalyze the data reported in this paper is available from the lead contact upon reasonable request.

### Experimental model and study participant details

#### Subjects and specimens

Serum samples from individuals with PCR-confirmed SARS-CoV-2 infection were collected for routine laboratory testing between March 2021 and February 2022 during hospitalization at the University Medical Center, Freiburg (n = 13). Sera were also obtained from vaccinees receiving one (n = 5), two (n = 10), or three (n = 10) doses of Pfizer-BioNTech BNT162b2 mRNA vaccine. Serum samples from anonymous healthy controls (HD) who tested negative for SARS-CoV-2 IgG were used as controls in this study (n = 8). Demographic and clinical characteristics of the cohort of individuals are listed in Table S3.

#### Ethical statement

The protocol of this study conforms to the ethical guidelines of the 1975 Declaration of Helsinki and was approved by the institutional ethics committee of the University of Freiburg (EK 153/20). Written informed consent was obtained from participants, and the study was conducted according to federal guidelines and local ethics committee regulations (Albert-Ludwigs-Universität, Freiburg, Germany: No. F-2020-09-03-160428, No. 322/20; and No 20-1271_1). No compensation was given to research participants.

### Method details

#### Molecular cloning

Expression constructs anchoring the RBD were based on the tANCHOR display system for peptide or protein on the surface of human cells.^21^ To generate RBD variants, we used published data from the GISAID database.^50^ In detail, the vector ptANCHOR-CD82-V5-His-mCherry (ATG:biosynthetics GmbH, Merzhausen, Germany) was digested with *Eco*RI-HF and *Eco*RV-HF (NEB, New England Biolabs, Frankfurt, Germany) and ligated with T4 ligase (NEB) with *Eco*RI/*Eco*RV-digested RBD fragments (Table S1). DNA coding for the indicated RBD was produced by gene synthesis (ATG:biosynthetics GmbH). After incubation overnight at 4°C, the ligated product was transformed into chemocompetent *E. coli* DH5α (NEB). Plasmids were recovered from grown colonies using a Miniprep Kit (Qiagen, Hilden, Germany) and analyzed by restriction analysis (*Eco*RI, *Eco*RV) and Sanger sequencing. In the same way, a DNA insert coding for the ACE2_1-740_-V5-His protein (Table S1) was codon optimized and cloned into the vector FlexMam-Puro (ATG:biosynthetics GmbH) using the restriction sites *Nhe*I/*Pme*I. In order to generate the Wuhan RBD tANCHOR construct with the YFP fusion (ptANCHOR-CD82-Wuhan-YFP), the insert coding for YFP was amplified by Q5 hotstart high-fidelity DNA polymerase (NEB) using the primer YFP-*Cla*I for/YFP-*Pme*I rev (Table S2, Integrated DNA Technologies, Leuven, Belgium) and the template vector pCMV-CD63-YFP.^49^^,^^51^ The amplified YFP fragment was inserted into the ptANCHOR-CD82-Wuhan-mCherry vector by exchanging the mCherry coding sequence with the YFP using the restriction sites *Cla*I/*Pme*I. Plasmid DNA for transfection of HeLa and HEK293Tcells was produced using the Maxiprep kit (Qiagen). When producing vectors for the neutralization assay, the plasmid DNA was diluted with water to reach a stock concentration of 500 ng/μL +/− 20 ng/μL for all RBD tANCHORed constructs. Plasmid DNA concentration was measured by using a NanoVue spectrophotometer (GE HealthCare, Solingen, Germany) and quality was further checked by linearization of 1 μg plasmid DNA with *Eco*RI-HF (NEB) for 1 h and analyzed by 1% agarose (Thermo Fisher scientific) gel electrophoresis (Figure S2).

#### Cell culture and cell-based SARS-CoV-2 ELISA

HeLa or HEK293T cells were maintained in Dulbecco’s modified Eagle’s medium (DMEM); supplemented with 2 mM L-glutamine, 10% fetal bovine serum (FBS, Gibco), 100 U/mL penicillin, and 100 μg/mL streptomycin; and incubated at 37°C in a 5% CO_2_ humidified atmosphere. Cells were detached from the culture flask with trypsin-EDTA and, after washing with 1X PBS, counted using an Assistent Neubauer-improved cell counting chamber (Hecht Glaswarenfabrik, Sondheim vor der Rhön, Germany). HeLa cells (1.5 x 10^4^) were seeded in a 96-well cell culture plate (TPP). After cells reached ∼80% confluency, they were transfected with 0.3 μg or the indicated amount of plasmid DNA, and 1 μL Metafectene (Biontex, Munich, Germany) per well of a 96-well plate. DNA and transfection reagent were solved separately in 50 μL of serum-free medium, mixed by one-time pipetting, and incubated for 20 min at room temperature. After that, the transfection mix was added to the cells (final volume of 100 μL). Cells were incubated for 24 h, and the medium was replaced by 100 μL of fresh medium containing 10% FBS and incubated for a further 24 h, washed once with 1X PBS, and fixed for 20 min with 2% paraformaldehyde (PFA) in 1X PBS, followed by two washing steps with 300 μL of 1X PBS. Cells were blocked for 2 h with freshly prepared blocking 1X PBS buffer containing 2% chicken albumin (Sigma Aldrich, Steinheim, Germany), 3% BSA fraction V (Carl Roth, Karlsruhe, Germany), and 10% normal goat serum (Biowest, Nuaillé, France). Cells were then incubated with diluted sera in blocking buffer for 1 h, washed in a single wash cycle with 1X PBS containing 0.05% Tween 20 (Sigma Aldrich) using the ELISA washer BioTec 405 (Agilent Technologies, Waldbronn, Germany) with a flow rate of 3. The washing volume was 450 μL, and the delay time was 30 s. Subsequently cells were incubated with 100 μL (or the indicated amount) of supernatant collected from cells secreting ACE2_1-740_-V5-His protein for 1.5 h. After incubation with the ACE2_1-740_-V5-His protein cells were washed with three washing cycles and incubated with 100 μL of mouse anti-V5-HRP 1:8,000 (1.18 mg/mL, Invitrogen, Thermo Fisher Scientific, Germany) for 45 min. Cells were washed with five washing cycles and HRP-bound antibodies were detected with 80 μL TMB solution (Bio-Rad, Munich, Germany). The HRP enzyme reaction was stopped with 100 μL of 2 M H_2_SO_4_. Absorbance was measured at a wavelength 450 nm with the reference at 620 nm (450 nm–620 nm) using a TECAN spectrophotometer Infinite 200 (Tecan, Crailsheim, Germany). Neutralizing activity was calculated using the Equation 1 (Equation 1):

(Equation 1) $$\%\;\text{Neutralizing}\;\text{activity}=\left(1-\;\frac{A_{\text{sample}}}{A_{\text{total}}}\right)*100$$

Where A_sample_ is the measured absorbance value for cells incubated with serum, and A_total_ is the measured absorbance value for cells incubated with blocking buffer without serum. The A_total_ value represents the maximum ACE2_1-740_-V5-His binding that can be achieved. Background binding (mock transfected) was subtracted from all values.

#### Monitoring of cell adherence during washing steps

To test for cell adhesion and cell loss, a confluent 96-well plate of HeLa and HEK293T cells, respectively, was fixed with 2% PFA in 1X PBS for 20 min, washed with 1X PBS, and attached cells were stained with crystal violet solution containing 1% crystal violet in 20% methanol (Carl Roth, Karlsruhe, Germany) for 30 min. After staining, crystal violet dye was washed out with pure water, and the washing test was performed by using an ELISA washer. Cell adhesion and cell loss was imaged by using a ZEISS microscope Axiovert 40 CFL with a 20x objective and a numerical aperture of 0.30 with implemented color AxioCam ICc1. To capture the bottom of the 96-well plate, it was placed on a Reflecta light plate (Intas, Göttingen, Germany), and the well bottom was imaged by using a SONY NEX5 digital camera with a SONY macro E3.5/30 objective (Sony, Tokyo, Japan). In order to analyze cell adherence of transfected cells the wells were imaged using an AxioObserver fluorescence microscope with an Axiocam 503 mono, filter cube for red dyes and a 20x objective (Zeiss).

#### Nucleocapsid protein (NCP)-specific ELISA

Covid-19 uninfected individuals in the groups receiving one, two, or three doses of Pfizer-BioNTech BNT162b2 mRNA vaccine were confirmed by reactivity test to nucleocapsid protein (NCP) conducted in parallel to cell-based ELISA and performed by using the same starting dilution of incubated sera. We coated 96-well microplates Nunc MaxiSorp (Thermo Fisher Scientific) with 100 μL of 0.5 μg/mL SARS-CoV-2 NCP (amino acids 2–419, Miltenyi Biotec, Bergisch Gladbach, Germany) diluted in coating buffer containing 15 mM Na_2_CO_3_ (Merck, Darmstadt, Germany) and 35 mM NaHCO_3_ (Merck, Darmstadt, Germany) at pH 9.6 and sealed with ROTILABO (Carl Roth) seal film overnight at 4°C. The 96-well microplates were then washed three times with 1X PBS using an ELISA washer, blocked with 100 μL blocking buffer containing 2% chicken albumin (Sigma Aldrich) and 3% BSA fraction V (Carl Roth) for 30 min, and then washed three times with 1X PBS and incubated with 100 μL of diluted sera 1:200 in blocking buffer containing 10% normal goat serum (NGS). After 1 h, the plates were washed five times with 450 μL water containing 0.05% Tween 20, and after the washing step, 100 μL of rabbit anti-human-HRP antibody 1:5,000 (1.3 mg/mL, DAKO) diluted in blocking buffer containing 10% NGS was added. After incubation with the secondary antibody, the 96-well microplates were washed five times with 450 μL water containing 0.05% Tween 20. Bound HRP-conjugated secondary antibodies were detected with 80 μL of TMB (3,3′,5,5′-tetramethylbenzidine) substrate (BioRad, Munich, Germany) by incubation at room temperature for 5 min, and the reaction was stopped by addition of 100 μL of 2 M H_2_SO_4_. Absorbance values were measured at a wavelength of 450 nm with background correction at 620 nm by using a microplate reader Infinite 2000 (Tecan). Cut-off values were calculated using the mean + 3 standard deviations (SDs) derived from negative controls.

#### Colocalization of variant RBD compared to the Wuhan variant

HeLa cells (1 × 10^4^) were seeded into each well of a high glass-bottomed eight well IBIDI μ-slide (Ibidi, Munich, Germany) and after 24 h were transiently transfected with 0.3 μg of each of the indicated plasmids and 1 μL Metafectene like described in the cell-based SARS-CoV-2 ELISA section. After 48 h, cells were fixed with 200 μL paraformaldehyde in 1X PBS for 20 min. The PFA solution was removed, and 200 μL 1X PBS was added to each well. Images were acquired using an inverted confocal laser scanning microscope (LSM 780; Carl Zeiss Microscopy GmbH, Oberkochen, Germany) and a plan-apochromat oil immersion objective 63×, numerical aperture 1.4 (Carl Zeiss Microscopy GmbH, Oberkochen, Germany). Fluorescence signals were detected with the Zeiss ZEN smart setup settings for mCherry and YFP fluorescence proteins. Pearson’s correlation coefficient (PCC) was calculated using the Fiji ImageJ Coloc2 plugin.

#### Detection of bound ACE2_1-740_-V5-His by confocal laser scanning microscopy (CLSM)

HeLa cells (1 × 10^4^/well) were seeded into a high glass-bottomed eight-well IBIDI μ-slide (Ibidi, Munich, Germany) and were transiently transfected with 0.3 μg plasmid DNA and 1 μL Metafectene like described in the cell-based SARS-CoV-2 ELISA section. After 48 h post-transfection, cells were washed once with 1X phosphate-buffered saline (PBS) and fixed with 200 μL 2% PFA in 1X PBS for 20 min. Cells were washed two times with 200 μL 1X PBS and left for 1 h in 200 μL blocking buffer containing 10% normal goat serum. After the blocking step cells were incubated for 1 h with 200 μL of supernatant containing ACE2_1-740_-V5-His protein (corresponds to 1.4 μg protein), washed three times with 200 μL 1X PBS and bound ACE2_1-740_-V5-His was detected by incubation of 200 μL of rabbit anti-V5 antibody (1 mg/mL, Novus Biologicals, Littleton, United States) diluted (1:2,000) in blocking buffer containing 10% normal goat serum for 1 h at room temperature. Cells were washed three times with 1X PBS, and to detect bound rabbit anti-V5 antibodies, cells were incubated with goat anti-rabbit-Alexa 488 (2 mg/mL, Invitrogen, Thermo Fisher Scientific) at a dilution of 1:3,000 in 3% BSA for 1 h. After three washing steps, cells were left in 200 μL 1X PBS in the presence of 1 μL of Hoechst 33342 ready to use staining solution (Immunochemistry technologies, CA, USA). Images were acquired using an inverted confocal laser scanning microscope and a 63× plan-apochromat oil immersion objective with a numerical aperture of 1.4. Fluorescence signals and were detected with the Zeiss ZEN smart setup settings for mCherry, Hoechst 33342, and Alexa 488 dyes.

#### Secretion of ACE2_1-740_-V5-His protein

HEK293T cells (6-well format) were transfected at a confluency of ∼80% with 4 μg plasmid DNA and 8 μL Metafectene (Biontex, Munich, Germany) according to the manufacturer’s instructions. After 24 h, the medium was replaced by fresh medium containing 5 μg/mL puromycin (Carl Roth). Cells were treated after 3 days with a lower concentration of 2 μg/mL until enough viable cells were obtained from a 6-well plate. Cells were then detached with trypsin (Sigma Aldrich) and cultivated stepwise in larger flasks (25, 75, 150 mL) until enough cells were harvested for a 300 mL flask. Cells were harvested in a 300 mL flask until a confluency of 80% was reached, the medium was replaced, and 50 mL of medium was added. After 3 days, the supernatant was harvested, centrifuged to remove cell debris, pooled with another collected supernatant, and stored at −80°C until use. We used the same supernatant batch to perform all neutralizing assays.

#### Expression control and characterization of secreted ACE2_1-740_-V5-His protein by Western blot analysis

One milliliter of supernatant from HEK293T cells transiently or stably transfected with the construct pACE2_1-740_-V5-His in a 6-well format was collected and centrifuged in order to separate cell debris from supernatants. Cleared supernatants were adjusted to pH 8.0 with 2 M NaOH solution (1–5 μL) and were added to 50 μL of a slurry of His tag isolation Dynabeads (Invitrogen) and incubated for 1 h at 4°C. Dynabeads were washed two times with wash buffer (1X PBS containing 0.05% Tween 20), and after magnetic separation, Dynabeads were eluted with 10 μL of elution buffer adjusted to pH 8.0, containing 300 mM imidazole (Carl Roth), 300 mM NaCl (Carl Roth), 50 mM Na_3_PO_4_ (Sigma Aldrich), and 0.01% Tween 20. Eluted proteins were diluted with 20 μL of 2x Laemmli buffer. Cells were lysed in 100 μL 2x Laemmli buffer (Sigma Aldrich) containing 25 U of Benzonase (Millipore) and 1 μL HALT protease inhibitor cocktail (Thermo Fischer Scientific) in order to obtain cell lysates. After DNA degradation (3–5 min), His-tagged protein extracts were boiled for 3 min at 95°C and separated by SDS-PAGE using 4%–15% Protean TGX gels (Bio-Rad, Munich, Germany) and 1X SDS-PAGE buffer for electrophoresis. SDS-PAGE buffer (10x) contains 144.4 g Glycine (Carl Roth), 30.3 g Tris (Carl Roth), and 10 g SDS pellets (Carl Roth). Separated proteins were transferred to a 0.2-μm PVDF Mini *trans*-blot membrane (Bio-Rad) and then blocked for 30 min with 10% low-fat milk powder (Carl Roth) diluted in pure water. Proteins were detected using the specific primary antibodies 1:10,000 mouse anti-V5-HRP (1.18 mg/mL, Invitrogen), 1:10,000 anti-beta-actin-HRP (1 mg/mL, Thermo), or 1:5,000 rabbit anti-ACE2 (1 mg/mL, Invitrogen) with a 1-h incubation time. Secondary goat anti-rabbit-HRP (0.25 g/mL, Dako, Agilent Technologies, Denmark) was used at a dilution of 1:10,000 and incubated for 45 min. The activity of HRP was detected with Pierce West Pico substrate (Thermo Fisher scientific) and the Chemocam digital image analyzer (Intas, Göttingen, Germany).

#### Quantification of secreted ACE2_1-740_-V5-His protein

A 20-μL volume of V5-NanoTrap magnetic agarose slurry (Proteintech Germany GmbH, Planegg-Martinsried, Germany) was washed once with 200 μL 1X PBS and incubated for 2 h at 4°C with 500 μL or 250 μL of supernatant collected from ACE2_1-740_-V5-His-secreting HEK293T cells. After incubation, magnetic agarose beads were washed three times with 500 μL 1X PBS 0.05% Tween 20. After the first wash, magnetic agarose beads were transferred to a new 1.5-mL reaction tube. Ten microliters of 2x Laemmli buffer was added to magnetic agarose beads, heated for 3 min at 90°C, and separated by SDS-PAGE. As a control, 500 μL of supernatant from non-transfected HEK293T cells was treated in the same way. In order to generate a BSA standard curve, Pierce BSA standard 2 mg/mL was diluted in 2x Laemmli buffer to obtain 0.25, 0.5, 1, 2, and 4 μg BSA protein per lane. Lysed protein samples were separated by SDS-PAGE as described above. The gel was stained with Coomassie dye containing 0.1% Coomassie Brilliant Blue R-250 (Carl Roth), 50% methanol (Carl Roth), 10% glacial acetic acid (Carl Roth), and 40% water for 1 h, and the gel was de-stained with 50% water, 40% methanol, and 10% glacial acetic acid. Visualized protein bands representing ACE2_1-740_-V5-His were quantified by generating a standard curve based on the integrated density protein band values of the BSA standard. For this, the de-stained gel was photographed (SONY NEX5 camera) and processed using the ImageJ gel analyzer plugin.

#### Quantification of mCherry reporter protein

Expressed mCherry reporter protein was quantified by using the mCherry quantification kit (Abcam, Amsterdam, Netherlands). HeLa cells were transfected with different SARS-CoV-2 variants in a 96-well format in the same way as described above for the cell-based SARS-CoV-2 ELISA. In detail, HeLa cells were lysed after 48 h post-transfection in 35 μL mCherry assay buffer and were incubated on ice for 15 min. Cell lysates from three wells were collected in a 1.5-mL reaction tube and centrifuged in order to remove cell debris, and 100 μL supernatants were transferred to a 96-well optical plate (Greiner Bio-One, Frickenhausen, Germany). In addition, mCherry standard protein provided with the mCherry quantification kit was used to generate a standard curve (diluted in mCherry assay buffer to obtain 0, 20, 40, 60, 80, 100, and 120 ng/well) from the fluorescence readings to calculate the amount of mCherry protein that was extracted from HeLa cells. All fluorescence values were measured using the GloMax-Multi+ detection system in combination with the filter kit green for red dyes (Promega). The relative light unit (RLU) value measured for wells containing only mCherry assay buffer was subtracted from all readings.

#### Quantification of mCherry fluorescence from epifluorescence images

Transfection of HeLa cells was performed as described in the section on the cell-based SARS-CoV-2 ELISA. HeLa cells expressing tANCHORed RBD variants were captured in a 96-well format by an inverted AxioObserver fluorescence microscope with a Axiocam 503 mono, filter cube for red dyes and a 20x objective (Zeiss). All epifluorescence images were analyzed with Fiji ImageJ software in order to obtain mean fluorescence values for mCherry signals. From each well, three images were taken for analysis, and the mean value was used as one data point.

#### NeutraLISA for analysis of SARS-CoV-2 neutralization activity

SARS-CoV-2 neutralization activity of antibodies was measured with the NeutraLISA system (EUROIMMUN, Lübeck, Germany) following the instructions of the manufacturer. Serum samples were diluted 1:5 and 1:20 in sample buffer containing biotinylated ACE2. Inhibition values (%) were calculated (Equation 1) from absorbance values at 450 nm and reference wavelength 620 nm with an Infinite 200 microplate reader (Tecan). Cut-off values are defined by the manufacturers as follow: <20%: negative; between ≥20% and <35%: borderline; ≥35%: positive.

### Quantification and statistical analysis

The Student's unpaired *t* test was used to compare two groups and one-way ANOVA to test for differences among more than two groups. A p value less than 0.05 was considered as statistically significant, non-significant (ns): p > 0.05, ∗: p < 0.05, ∗∗: p < 0.01, ∗∗∗: p < 0.001, ∗∗∗∗: p < 0.0001. Data are presented as mean ± standard deviation (SD).

### Additional resources

There are no additional resources to be reported.
