# Supplementary material for: tANCHOR-cell-based assay for monitoring of SARS-CoV-2 neutralizing antibodies rapidly adaptive to various receptor-binding domains
Source: iScience. 2024 Feb 5;27(3):109123. doi: 10.1016/j.isci.2024.109123 (PMC10877956; doi:10.1016/j.isci.2024.109123)
Supplement: Document S1. Figures S1 and S2 and Tables S1–S3 [file mmc1.pdf]

iScience, Volume *27*

Supplemental information

tANCHOR-cell-based assay for monitoring of SARS-CoV-2 neutralizing antibodies rapidly adaptive to various receptor-binding domains

Daniel Ivanusic, Josef Maier, Suheda Icli, Valeria Falcone, Hubert Bernauer, and Norbert Bannert

Figure S1: Representative confocal laser scanning microscopy (CLSM) images from colocalization experiments, related to Figure 1. HeLa cells were transiently transfected with the indicated plasmid DNA. The calculated Pearson correlation coefficient (PCC) is given on the merged image. All scale bars, 10 μm.

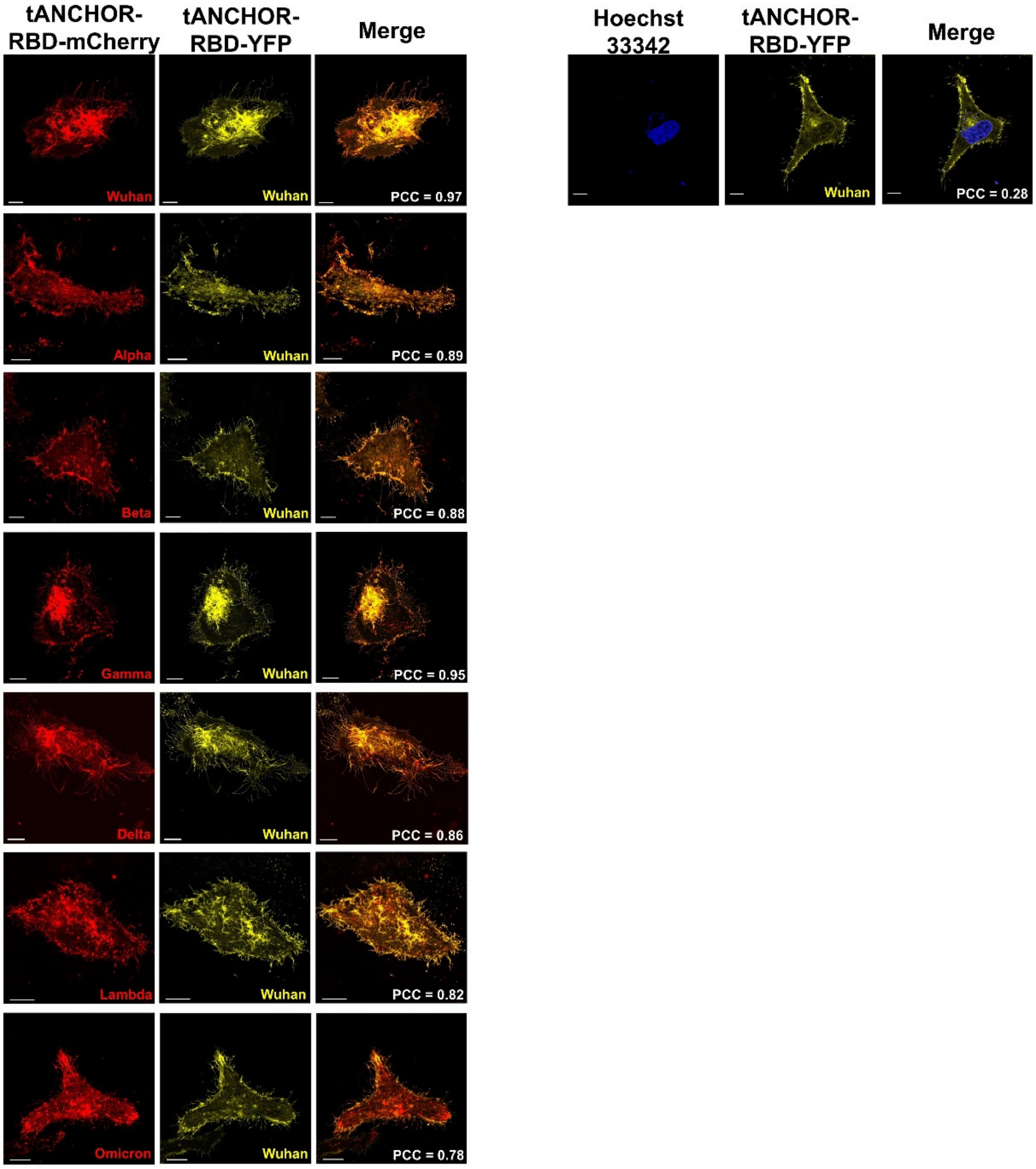

Figure S2: Quality check of isolated plasmid DNA, related to STAR★Methods. Separation of linearized plasmid DNA (1 µg) with *Eco*RI-HF by using a 1% agarose gel containing ethidium bromide

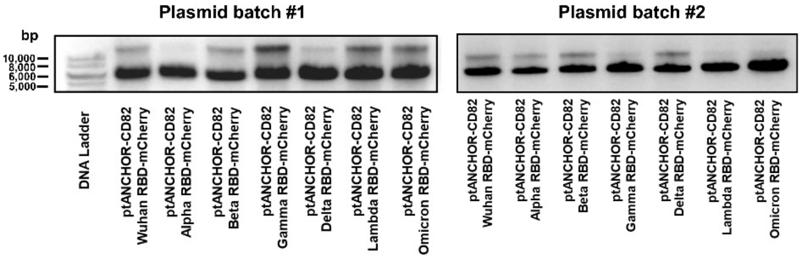

Supplemental Table 1: Sequence of gene synthesis fragment used for cloning, related to STAR★Methods.

SARS-CoV-2 Wuhan-Hu-1 RBD gene synthesis fragment GenBank accession: NC_045512.2:22517-23191

```
GAATTCAGAGTCCAACCAACAGAATCTATTGTTAGATTTCCTAATATTACAAACTTGTGCCCTTTTGGTGAAGTTTTTAACGCCACCAGATTTGCATCTGTTTATGCTT
GGAACAGGAAGAGAATCAGCAACTGTGTTGCTGATTATTCTGTCCTATATAATTCCGCATCATTTTCCACTTTTAAGTGTTATGGAGTGTCTCCTACTAAATTAAATGA
TCTCTGCTTTACTAATGTCTATGCAGATTCATTTGTAATTAGAGGTGATGAAGTCAGACAAATCGCTCCAGGGCAAACTGGAAAGATTGCTGATTATAATTATAAATTA
CCAGATGATTTTACAGGCTGCGTTATAGCTTGGAACTCTAACAATCTTGATTCTAAGGTTGGTGGTAATTATAATTACCTGTATAGATTGTTTAGGAAGTCTAATCTCA
AACCTTTTGAGAGAGATATTTCAACTGAAATCTATCAGGCCGGTAGCACACCTTGTAATGGTGTTGAAGGTTTTAATTGTTACTTTCCTTTACAATCATATGGTTTCCA
ACCCACTAATGGTGTTGGTTACCAACCATACAGAGTAGTAGTACTTTCTTTTGAACTTCTACATGCACCAGCAACTGTTTGTGGACCTAAAAAGTCTACTAATTTGGTT
AAAAACAAATGTGTCAATTTCAACTTCGATATC
```

SARS-CoV-2 Wuhan-Hu-1 RBD protein sequence GenBank accession: YP_009724390.1:318-543

```
EFRVQPTESIVRFPNITNLCPFGEVFNATRFASVYAWNRKRISNCVADYSVLYNSASFSTFKCYGVSPTKLNDLCFTNVYADSFVIRGDEVRQIAPGQTGKIADYNYKL
PDDFTGCVIAWNSNNLDSKVGGNYNYLYRLFRKSNLKPFERDISTEIYQAGSTPCNGVEGFNCYFPLQSYGFQPTNGVGYQPYRVVVLSFELLHAPATVCGPKKSTNLV
KNKCVNFNFDI
```

Note: Underlined sequence contains the flanked restriction sites *Eco*RI and *Eco*RV. *Eco*RI is coding for EF and therefore F is part of the RBD that is corresponding to amino acid position 318 GenBank: YP_009724390.1.

Sequence for construction of Alpha, Beta, Gamma, Delta, Lambda and Omicron RBD variants

Data Availability

GISAID Identifier: EPI_SET_230215my

doi: 10.55876/gis8.230215my

All genome sequences and associated metadata in this dataset are published in GISAID's EpiCoV database. To view the contributors of each individual sequence with details such as accession number, Virus name, Collection date, Originating Lab and Submitting Lab and the list of Authors, visit 10.55876/gis8.230215my

Data Snapshot
EPI_SET_230215my is composed of 9 individual genome sequences.The collection dates range from 2019-12-30 to 2021-11-20; Data were collected in 7 countries and territories; All sequences in this dataset are compared relative to hCoV-19/Wuhan/WIV04/2019 (WIV04), the official reference sequence employed by GISAID (EPI_ISL_402124). Learn more at https://gisaid.org/WIV04.

			Position in genome	
**SARS-CoV-2 variant**	**GISAID EPI_ISL**	**Strain**	**Start**	**End**
Wuhan-Hu-1	EPI_ISL_402124.1	hCoV-19/Wuhan/WIV04/2019	22517	23191
Alpha B.1.1.7	EPI_ISL_601443.1	hCoV-19/England/MILK-9E05B3/2020	22445	23119
Beta B.1.351	EPI_ISL_736932.1	hCoV-19/South_Africa/KRISP-BH02956385/2020	22496	23170
Beta B.1.351	EPI_ISL_803957.1	hCoV-19/Germany/NW-RKI-I-0029/2020	22492	23166
Gamma P.1	EPI_ISL_792681.1	hCoV-19/Japan/IC-0562/2021	22473	23147
Delta B.1.617.2	EPI_ISL_2378732.1	hCoV-19/Japan/TKYTK1734/2021	22507	23181
Lambda C.37	EPI_ISL_14204502.1	hCoV-19/Brazil/SP-IB_RP82131/2021	22470	23144
Omicron BA.1	EPI_ISL_6640916.1	hCoV-19/Botswana/R40B59_BHP_3321001248/2021	22442	23116
Omicron BA.1	EPI_ISL_6704867.1	hCoV-19/South_Africa/NICD-N21668/2021	22493	23167

Note: The RBD coding sequence is 675 base pairs long and encodes 225 amino acids. It represents the amino acids 319-543 of the spike protein (according to Wuhan-1 numbering). For both, Beta and Omicron BA-1, two type strains were respectively considered with the same outcome. The RBD coding sequences of the two Beta and the two Omicron BA.1 strains are identical to each other, respectively. The *Eco*RI restriction recognition sites at position 354-359 GAATTC, with positions counted from the start of codon F318, present within the RBD-coding sequences in all strains, were removed by the silent base change 357TC. N-terminal *Eco*RI is coding for EF and therefore F is part of the RBD that is corresponding to amino acid position 318.

**Variant**	**Mutation sites within the RBD**
Alpha B.1.1.7	N501Y
Beta B.1.351	K417N, E484K, N501Y
Gamma P.1	K417T, E484K, N501Y
Delta B.1.617.2	L452R, T478K
Lambda C.37	L452Q, F490S
Omicron BA.1	G339D, S371L, S373P, S375F, K417N, N440K, G446S, S477N, T478K, E484A, Q493R, G496S, Q498R, N501Y, Y505H

Human angiotensin-converting enzyme 2 (ACE2) gene synthesis fragment
Sequence was fused with coding sequence for V5 and 6 x His tag

GCTAGCAATTTATAACGAGA**ATGAGCAGCAGCAGCTGGCTGCTGCTGAGCCTGGTGGCCGTGACCGCCGCCCAGAGCACCATCGAGGAGCAGGCCAAGACCTTCCTGGA**
CAAGTTCAACCACGAGGCCGAGGACCTGTTCTACCAGAGCAGCCTGGCCAGCTGGAACTACAACACCAACATCACCGAGGAGAACGTGCAGAACATGAACAACGCCGGC
GACAAGTGGAGCGCCTTCCTGAAGGAGCAGAGCACCCTGGCCCAGATGTACCCCCTGCAAGAGATCCAGAACCTGACCGTGAAGCTGCAACTGCAAGCCCTGCAACAGA
ACGGCAGCAGCGTGCTGAGCGAGGACAAGAGCAAGCGCCTGAACACCATCCTGAACACCATGAGCACCATCTACAGCACCGGCAAGGTGTGCAACCCCGACAACCCCCA
GGAGTGCCTGCTGCTGGAGCCCGGCCTGAACGAGATCATGGCCAACAGCCTGGACTACAACGAGCGCCTGTGGGCCTGGGAGAGCTGGCGCAGCGAGGTGGGCAAGCAG
CTGCGCCCCCTGTACGAGGAGTACGTGGTGCTGAAGAACGAGATGGCCCGCGCCAACCACTACGAGGACTACGGCGACTACTGGCGCGGCGACTACGAGGTGAACGGCG
TGGACGGCTACGACTACAGCAGGGGCCAGCTGATCGAGGACGTGGAGCACACCTTCGAGGAGATCAAGCCCCTGTACGAGCACCTGCACGCCTACGTGCGCGCCAAGCT
GATGAACGCCTACCCCAGCTACATCAGCCCCATCGGGTGCCTGCCCGCCCACCTGCTGGGCGACATGTGGGGCCGCTTCTGGACCAACCTGTACAGCCTGACCGTGCCC
TTCGGCCAGAAGCCCAACATCGACGTGACCGACGCGATGGTGGACCAGGCCTGGGACGCCCAGCGCATCTTCAAGGAGGCCGAGAAGTTCTTCGTGAGCGTGGGCCTGC
CCAACATGACCCAGGGCTTCTGGGAGAACAGCATGCTGACCGACCCCGGCAACGTGCAGAAGGCCGTGTGCCACCCCACCGCCTGGGACCTGGGCAAGGGCGACTTCCG
CATCCTGATGTGCACCAAGGTGACTATGGACGACTTCCTGACCGCCCACCACGAGATGGGCCACATCCAGTACGACATGGCCTACGCCGCCCAGCCCTTCCTGCTGCGC
AACGGCGCCAACGAGGGCTTCCACGAGGCCGTGGGCGAGATCATGAGCCTGAGCGCCGCCACCCCCAAGCACCTGAAGAGCATCGGCCTGCTGAGCCCCGACTTCCAGG
AGGACAACGAGACCGAGATCAACTTCCTGCTGAAGCAGGCCCTGACCATCGTGGGCACCCTGCCCTTCACCTACATGCTGGAGAAGTGGCGCTGGATGGTGTTCAAGGG
CGAGATCCCCAAGGACCAGTGGATGAAGAAGTGGTGGGAGATGAAGCGCGAGATCGTGGGCGTGGTGGAGCCCGTGCCCCACGACGAGACCTACTGCGACCCCGCCAGC
CTGTTCCACGTGAGCAACGACTACAGCTTCATCCGCTACTACACCCGCACCCTGTACCAGTTCCAGTTCCAGGAGGCCCTGTGCCAGGCCGCCAAGCACGAGGGTCCCC
TGCACAAGTGCGACATCAGCAACAGCACCGAGGCCGGCCAGAAGCTGTTCAACATGCTGCGCCTGGGCAAGAGCGAGCCCTGGACCCTGGCCCTGGAGAACGTGGTGGG
CGCCAAGAACATGAACGTGCGCCCCCTGCTGAACTACTTCGAGCCCCTGTTCACCTGGCTGAAGGACCAGAACAAGAACAGCTTCGTGGGCTGGAGCACCGACTGGAGC
CCCTACGCCGACCAGAGCATCAAGGTGCGCATCAGCCTGAAGAGCGCCCTGGGCGACAAGGCCTACGAGTGGAACGACAACGAGATGTACCTGTTCCGCAGCAGCGTGG
CCTACGCCATGCGCCAGTACTTCCTGAAGGTGAAGAACCAGATGATCCTGTTCGGCGAGGAGGACGTGCGCGTGGCCAACCTGAAGCCCCGCATCAGCTTCAACTTCTT
CGTGACCGCCCCCAAGAACGTGAGCGACATCATCCCCCGCACCGAGGTGGAGAAGGCCATCCGCATGAGCCGCAGCCGCATCAACGACGCCTTCCGCCTGAACGACAAC
AGCCTGGAGTTCCTGGGCATCCAGCCCACCCTGGGTCCCCCCAACCAGCCCCCCGTGAGCGGAAAGGGCCCGCGGTTCGAAGGTAAGCCTATCCCTAACCCTCTCCTCG
GTCTCGATTCTACGCGTACCGGTCATCATCACCATCACCATTGAGTTTAAAC

Note: DNA sequence coding for ACE2 amino acids 1-740 is bold displayed, sequence coding for the V5-6xHis tag is underlined, other sequence 5´and 3´ is used for cloning in the target vector using the restriction sites *Nhe*I/*Pme*I and the linker to clone sequence in frame.

Angiotensin-converting enzyme 2 isoform 2 precursor [Homo sapiens]
GenBank RefSeq Protein: NP_001373188.1:1-740

MSSSSWLLLSLVAVTAAQSTIEEQAKTFLDKFNHEAEDLFYQSSLASWNYNTNITEENVQNMNNAGDKWSAFLKEQSTLAQMYPLQEIQNLTVKLQLQALQQNGSSVLS
EDKSKRLNTILNTMSTIYSTGKVCNPDNPQECLLLEPGLNEIMANSLDYNERLWAWESWRSEVGKQLRPLYEEYVVLKNEMARANHYEDYGDYWRGDYEVNGVDGYDYS
RGQLIEDVEHTFEEIKPLYEHLHAYVRAKLMNAYPSYISPIGCLPAHLLGDMWGRFWTNLYSLTVPFGQKPNIDVTDAMVDQAWDAQRIFKEAEKFFVSVGLPNMTQGF
WENSMLTDPGNVQKAVCHPTAWDLGKGDFRILMCTKVTMDDFLTAHHEMGHIQYDMAYAAQPFLLRNGANEGFHEAVGEIMSLSAATPKHLKSIGLLSPDFQEDNETEI
NFLLKQALTIVGTLPFTYMLEKWRWMVFKGEIPKDQWMKKWWEMKREIVGVVEPVPHDETYCDPASLFHVSNDYSFIRYYTRTLYQFQFQEALCQAAKHEGPLHKCDIS
NSTEAGQKLFNMLRLGKSEPWTLALENVVGAKNMNVRPLLNYFEPLFTWLKDQNKNSFVGWSTDWSPYADQSIKVRISLKSALGDKAYEWNDNEMYLFRSSVAYAMRQY
FLKVKNQMILFGEEDVRVANLKPRISFNFFVTAPKNVSDIIPRTEVEKAIRMSRSRINDAFRLNDNSLEFLGIQPTLGPPNQPPVSGKGPRFEGKPIPNPLLGLDSTRT
GHHHHHH

Note: Bold displayed protein sequence contains the amino acids of the ACE2 1-740 followed by the fused tags V5 and 6 x His that is underlined.

Supplemental Table 2: Sequences of oligonucleotide primers used for cloning, related to STAR★Methods.

Primer	5´→ 3´ Primer sequence
YFP-*Cla*I for	TTTTTAAGCTTATCGATGTGAGCAAGGGCGAGGAGCTG
YFP-*Pme*I	TTTTTGTTTAAACTCATGATCACTTGTACAGCTCGTCCATG

Supplemental Table 3: Demographic and clinical characteristics of the cohort, related to STAR★Methods.

Sample no.	Days after symptom onset	Age (years)	Sex male (m), female (f)
1	9	77	m
2	62	68	m
3	50	62	m
4	60	56	m
5	40	42	m
6	40	90	m
7	38	34	m
8	46	63	m
9	30	87	m
10	32	59	m
11	17	80	m
12	61	69	f
13	36	70	m
	**Number of vaccinations** (Pfizer-BioNTech BNT162b2)		
14	1	25	f
15	1	21	m
16	1	58	f
17	1	21	m
18	1	61	f
19	2	21	m
20	2	60	m
21	2	42	w
22	2	52	f
23	2	62	f
24	2	55	f
25	2	59	f
26	2	50	m
27	2	28	f
28	2	30	f
29	3	34	f
30	3	64	f
31	3	52	f
32	3	28	f
33	3	26	f
34	3	22	m
35	3	24	f
36	3	36	f
37	3	30	f
38	3	40	f

	COVID-19 negative control serum		
**39**	neg	56	f
**40**	neg	29	f
**41**	neg	56	f
**42**	neg	35	f
**43**	neg	64	f
**44**	neg	77	m
**45**	neg	42	m
**46**	neg	51	f
